# Assessment of Knowledge, Attitude, and Utilization of Traditional Medicine among the Communities of Debre Tabor Town, Amhara Regional State, North Central Ethiopia: A Cross-Sectional Study

**DOI:** 10.1155/2020/6565131

**Published:** 2020-03-10

**Authors:** Tezera Jemere Aragaw, Dessie Tegegne Afework, Kefyalew Ayalew Getahun

**Affiliations:** ^1^Department of Pharmacology, School of Pharmacy, College of Medicine and Health Sciences, University of Gondar, Gondar, Ethiopia; ^2^Department of Medical Laboratory, College of Health Sciences, Debre Tabor University, Debre Tabor, Ethiopia

## Abstract

**Background:**

Traditional medicine is used by about 80% of the Ethiopian people to meet their healthcare needs. The aim of this study was to assess the knowledge, attitude, and practice of the community on traditional medicine in Debre Tabor town.

**Methods:**

A community-based cross-sectional study was carried out from November 1, 2018, to December 30, 2018, with a face-to-face interview method and involved 402 participants recruited by systematic random sampling technique. Data were analyzed using SPSS version 20.0. The association of independent and dependent variables was determined by binary logistic regression.

**Results:**

Among the participants, 294 (73.13%) were females and 108 (26.87%) were males. The ages of participants ranged from 18 to 80 (mean age of the participants was 35.73 ± 0.59 years). Above three-fourths, 322 (80.1%) of the participants had good knowledge. 158 (39.3%) of the participants had a good attitude and 145 (36.1%) of the participants used traditional medicines in their lifetime for different ailments. From all the participants who use traditional medicine, 41 (28.3%) encountered minor adverse effects.

**Conclusions:**

The study participants in Debre Tabor have good knowledge but poor attitude and utilization of traditional medicine.

## 1. Introduction

A number of medicinal plants were in use as early as 5000 to 4000 BC in China, and 1600 BC by Syrians, Babylonians, Hebrews, and Egyptians [1]. Nowadays chemical and genetic constituents of plants are being increasingly exploited for the human benefit [2]. Current Studies indicate that 25% of modern medicines are derived from the extracts of medicinal plants [3]. About 80% of Ethiopian people rely on traditional medicine to meet their healthcare needs which could be attributed to cultural acceptability, perceived efficacy against certain types of diseases, physical accessibility, and affordability as compared to modern medicine [4]. Little effort was made to properly document the associated knowledge, attitude, and utilization of medicinal plants in the country [5]. Studies conducted hitherto are far from complete owing to multiethnic cultural diversity and the diverse flora of Ethiopia even thoug, encouraging initiatives have emerged in recent years [4, 6]. Ethiopia is often quoted as one of the six countries in the world where about 60% of plants are said to be indigenous with their healing potential [7–9]. Antibiotic research and development has slowed to a standstill due to market failure, lack of clear regulatory guidance for drug companies, and economic disincentives and are not as profitable compared to drugs used in chronic diseases [10]. Expenditures on medicines can represent up to 66% of total healthcare in developing countries and 50–90% of such expenditures are out-of-pocket expenses and 30% of the world's population and 50% of the poorest in Asia and Africa still lack access to essential medicines [11]. Essential medicine availability at the facility level was 91% based on a list of selected drugs vs. 84% based on prescriptions filled. From medicines prescribed less than half of them were obtained from the budget pharmacy, and 16.67% of patients were forced to purchase drugs in the private sector, where drugs are roughly twice as expensive [12]. Humans are dependent on other organisms for their life and the plant kingdom is the most essential to human well-being especially in terms of supplying his basic needs [13]. Indigenous knowledge is the accumulation of knowledge, rules, standards, skills, and mental sets, which are possessed by local people in a particular area [14, 15]. Prevention and elimination of physical, mental, or social imbalance can be treated by the combination of knowledge and practices of traditional medicine with exclusive practical experience and observation handed down from generation to generation, whether verbally or in writing [16]. Traditional medicine is becoming profitable, and multinational business is investing in it and billions of US dollars are being spent annually on it in many developed countries. About 32 billion dollars were spent in the United States of America on dietary supplements in 2012, an amount expected to increase to 60 billion dollars in 2021 [17]. The World Health Organization estimates about 83 billion US dollars global market of traditional medicine annually [18]. 33% to 50% of pharmaceutical medicines were originally derived from plants [19]. Digitalis, morphine, quinine, and vinca alkaloids are prominent examples that were obtained from plant sources [20]. Pharmacological research and drug development have relied on knowledge of traditional medicinal plants, not only when plant constituents are used directly as therapeutic agents, but also when they are used as starting materials for drug synthesis or as models for pharmacologically active compounds [21]. Plants have been used as a source of traditional medicine in Ethiopia to combat different ailments and human sufferings and due to its long period of practice and existence, traditional medicine has become an integral part of the culture of Ethiopian people [22, 23]. About 85 percent of the Ethiopian population does not have access to modern medicine. Thus, the development of medicinal plants in primary healthcare will not only save the foreign exchange but will also aid in conserving our national heritage [24]. In Ethiopia, medicinal plants are used in treatment of abscess, arthritis, ascariasis, burns, colds, colic, constipation, diabetes, dysentery, eclampsia, gastritis, gonorrhea, heartburn, headache, hemorrhoids, hepatitis, herpes simplex, kwashiorkor, leprosy, malaria, measles, rabbis, rheumatism, scabies, syphilis, schistosomiasis, and toothache [25]. The long history of the use of medicinal plants is reflected in various medicoreligious manuscripts produced on parchments and believed to have originated several centuries ago [26]. The fact that medical textbooks were written in Geez or even Arabic in Ethiopia between the mid-17^th^ and 18^th^ century implies that plants have been used as a source of traditional medicine in the Ethiopian healthcare system, for example, the use of *Hagenia abyssinica* to expel tapeworm and *Ruta chalepensis* for different health problems [27]. A cross-sectional study was done in Merawi town, where 61.5% of study participants had good knowledge about traditional medicines. 28.3% of the study participants prefer to use traditional medicines rather than modern health services. Traditional medicines are still accepted and counted on by 59.2% of the participants in the community and 49.5% of the participants agree that the reason is cultural acceptability. 42.2% of the participants after the use of traditional medicines had shown good outcome. Different types of herbal medicines were used by 70.9% of the participants either by themselves or through visiting traditional healers at least once in their lifetime. 22.7% of the participants had their family members experiencing adverse effects due to traditional medicine therapy [28]. From the study done in Nigeria contemporary community, 44.7% had knowledge about traditional medicine [29]. A study done in the Shirka district, Arsi Zone, revealed that 84% of traditional health practitioners supported the integration of modern medicine with traditional medicine to improve health care coverage in Ethiopia [30]. A cross-sectional study was done in Jara Town, Bale Zone, Southeast Ethiopia, where 96.3% of the respondents heard about traditional medicine, 43.91% of the respondents have planned to use traditional medicine in the future, 54.61% of the respondents believe that traditional medicines can cure diseases that are not cured by modern medicine, 63.6% suggest that herbal medicine users should consider herbal medicine is safe to use, 39.85% had positive attitude towards traditional medicine, 50.18% of the respondent accept traditional health practice, and 73.8% of the respondents have used traditional medicine at least once in their lifetime [31]. A cross-sectional study was done in Shopa Bultum, where 69.53% had knowledge about more than three types of traditional medicines, and 71.52% preferred traditional medicine for its affordability, accessibility, and acceptability. Medical herbalism was the most common traditional practice (79.47%). 35.76% of the respondents prefer to keep their knowledge as a secret. 72.85% of the respondents manage their acute/chronic illnesses by both self-medication and visiting traditional medicine practitioners, 36.42% of the respondents had good knowledge and of an older age and lower educational level. The percentage of males roughly increases from poor to good knowledge whereas that of the females decreases. 66.89% of the respondents were selecting both traditional medicine and modern medicine for curing illness and 79.47% of the respondents believe that traditional medicine can cure diseases better than modern doctors. 71.52% of the respondents prefer to visit traditional medicine practitioners first whenever they fall sick, and 71.52% of the respondents prefer traditional medicine to modern medicine due to affordability, accessibility, and acceptability. 72.85% of the respondents manage their acute/chronic illness by both self-medication and visiting traditional medicine practitioners [32].

About 80% of Ethiopian people rely on traditional medicine to meet their healthcare needs which could be attributed to cultural acceptability, perceived efficacy against certain types of diseases, physical accessibility, and affordability as compared to modern medicine. Little effort was made to properly document the associated knowledge, attitude, and utilization of medicinal plants in the country. Current studies indicate that 25% of modern medicines are derived from the extracts of medicinal plants. Studying the knowledge, attitude, and practices of traditional medicine in Debre Tabor town will provide important data for the town administration and Amhara Regional Health Bureau to take appropriate controlling measures regarding the quality and safety of the practices. The study will also provide the baseline data for the scientific society in written form which helps as a source for further investigations such as in vitro and in vivo studies, identification and molecular elucidation, and pharmacokinetic and pharmacodynamic profiles of secondary metabolites.

## 2. Method

### 2.1. Study Design and Study Period

A community-based cross-sectional study was conducted from November 1, 2016, to December 30, 2016, to assess knowledge, attitude, and utilization of the community towards TM, in Debre Tabor town, South Gondar administration zone of the Amhara Regional State, North Central Ethiopia.

### 2.2. Study Area

Debre Tabor town is located in the South Gondar administration zone of the Amhara Regional State, North Central Ethiopia, about 100 kilometers southeast of Gondar and 50 kilometers east of Lake Tana. This historic town has a latitude and longitude of 11°51′ (15.54 m) N38°1′ (30.48 cm) E with an elevation of 2,706 meters (8,877.95 ft) above sea level. Its climatic condition is “Dega.” [33]. Currently, it has a total population of 78,706 people of which 37683 (48%) are males and 41023 (52%) females and there are 12478 households in three “kebeles.”

### 2.3. Source Population

All the households in Debre Tabor town were the source population of the study.

### 2.4. Study Population

Individuals aged older than or equal to 18 years and living for at least six months in the town were included in the study. The sampling units were households, while the study units were adult individuals available in the household during the interview, preferably the females, if more than one adult was found in the household.

### 2.5. Sample Size Determination

Sample size was calculated based on the prevalence of knowledge, attitude, and practice from the following assumptions: *P*=80% which is the prevalence of TM users in Ethiopia [28], *Z* (1.96) is the value under standard normal table for confidence level of 95%, margin of error (*d*) = 4%, and using the formula for estimation of single population proportions *n* = *Z*^2^*P* (1 − *P*)/*d*^2^. = 385. Adding a nonresponse rate of 5% (19), the final sample size became 404 adults, where *n* is the required sample of the study.

### 2.6. Sampling Procedure

To select households, a systematic random sampling technique was used. The first household was selected from the list of initial 30 households by lottery method. Then every 31^st^ household was selected and adults in the household were interviewed. In the presence of more than one adult, the woman was interviewed as she took the highest responsibility in the care of family members. The husband or other adults have been interviewed in the absence of a woman.

### 2.7. Data Collection Procedure

Data were collected using structured interview administered questionnaire adapted from standardized questionnaires used by international organizations, national studies such as Ethiopian Demographic and Health Survey [34], and published articles in peer-reviewed journals [28–32]. Data collectors were briefed on the objective and relevance of the study on terms and how to collect the data using face-to-face interview.

#### 2.7.1. Data Quality Control

The data collection instruments were translated into the local language. A pilot test was done on 20 (5% of the sample population) in Gasay town which is 18 Kilometers away from Debre Tabor households to validate the consistency of the questions and data collection tool. The collected data was first being checked and cleaned for completeness.

### 2.8. Study Variables

The dependent variables of the study were knowledge, attitude, and utilization of the community on traditional medicines. The explanatory variables were the age of interviewee, educational status, religion, ethnicity, occupation, monthly family income, marital status, and family size.

### 2.9. Data Management and Analysis

Data were checked for completeness and consistency, were cleaned by the supervisor, entered into SPSS version 20.0 by the data clerk, and analyzed by investigators. The results were presented using simple frequencies with percentages in appropriate tables to display the descriptive part of the result. Six yes-or-no questions were asked to each respondent regarding knowledge and seven yes-or-no questions regarding attitude. The questions for which the respondent gave correct responses were counted and scored. This score was then pooled together and the mean score was computed to determine the overall knowledge of respondents; respondents who scored greater than or equal to the mean value were grouped as having good knowledge and attitude and those who scored less than the mean value were considered to be having poor knowledge and attitude.

### 2.10. Ethical Issues

Ethical clearance was requested and obtained from the Institutional Ethical Review Board of Debre Tabor University with a permission letter Ref. No:- DTU/RPD/121/2016 on 09/14/2016. Permission was requested to Debre Tabor town administration by a formal letter. Oral consent was asked from each participant of the study and the participants were informed that they can discontinue at any stage of the interview. All the participants who declared their willingness to participate were included in the study. The confidentiality of data was maintained by omitting their names and house numbers.

## 3. Results

### 3.1. Sociodemographic Characteristics

A total of 402 respondents, with a response rate of 99.5%, were studied. Among the participants, 294 (73.13%) were females and 108 (26.87%) were males. The ages of participants ranged from 18 to 80 (mean age of the participants was 35.73 ± 0.59 years). From the total respondents about 49 (12.2%) could not read and write, 40 (10%) could read and write, 28 (7%) had an educational level of grades 1–6, 27 (6.7) had an educational level of grades 7–12, 79 (19.7%) had a technical and vocational certificate, and 179 (44.5%) had a college diploma and above. Regarding the religion, 388 (96.4%) of the study participants were a follower of Orthodox Christianity, followed by Muslims, 14 (3.6%). 402 (100%) of the respondents had the Amhara nationality. Regarding the occupation of respondents, 162 (40.3%) were government employees, 83 (20.6%) were private-sector employees, 91 (22.6%) were housewives, 43 (10.7%) were merchants, and 23 (5.6%) were students. The average monthly incomes of study participants were 1953.80 ± 72.03 Birr, 247 (61.4%) of participants were from 100–2000 “Birr,” 132 (32.8%) were from 2001–3800 “Birr,” 18 (4.5%) were 3801–6000 “Birr,” and 5 (1.2%) were >6000 “Birr.” In terms of the marital status of participants, 298 (74.1%) were married, 56 (13.9%) were single, 36 (9.0%) were widowed, and 12 (3.0%) were separated/divorced. The average family sizes of the respondents were 3.23 ± 0.08 and from these 161 (40.0%) of the participants were 1–2, 154 (38.3%) of the participants were 3–4, 72 (17.9%) of the participants were 5–6, and 15 (3.7%) of the participants were >6 (See Table 1).

In the studied area, some aliments treated by TMs include abdominal cramp, abortion, burn, chest pain, cold, cough, cutaneous leishmaniasis, diarrhea, dry cough, fungal infection, gastritis, goiter, hemorrhoid, herpes zoster, hypertension, impotence, infected wound, joint pain, liver disease, malaria, myositis, rabies, scabies, tapeworm, tonsillitis, and toothache. Of these aliments, 90.91% were infectious diseases and the rest, 9.09%, were either acute or chronic noninfectious diseases. Leaves (55%), roots (20%), and seeds (8%) are majorly used to treat some of the aliments. In the studied area, the community used the different dosage forms from plants such as solid (48%), liquid (45%), and gas (7%). The plant materials were prepared in different dosage forms by drying, size reduction maceration, decoction, boiling, and direct compression of the fresh leaves and squeezing the liquid contents and were mainly used once daily for few days (ranged from 1 day to some months). The oral, topical, and inhalational routes were the most commonly used routes of administration. The locally available additives were used during preparation of traditional medicines (Table 5).

## 4. Discussion

From our study, the proportion of females in the sample was higher than males. This is somewhat expected due to the presence of females at the time of interview and involvement in family care. The results of this study revealed that overall practice of traditional medicine in the community is 35.8% which is lower than the previously reported studies in Shopa Bultum which is 79.47% [31], and this might be due to promotion and accessibility of modern medicine in the community even though traditional medicines have cultural acceptability, perceived efficacy against certain types of diseases, physical accessibility, and affordability as compared to modern medicine [4].

The result of this study revealed that overall knowledge of traditional medicine in the community is 80.1% which is higher than the previously reported studies in Shopa Bultum which is 69.53% [32]. This difference might be due to the age of the participants in our study, educational level, and religion of the respondents which slightly differ. Traditional medicine is the total combination of knowledge and practices that can be formally explained or used in the prevention and elimination of physical, mental, or social imbalance and relying exclusively on practical experience and observation handed down from generation to generation, whether verbally or in writing [16]. In the study area, the most widely known forms of traditional medicine were medical herbalism which is in line with previously reported studies [31]. Knowledge of traditional medicine is good among the elders and associated with the level of education of the communities of Debre Tabor town.

From the study, the majority of the participants 81.3% heard of traditional medicine and this percent is lower than a study done in Jara town which is 96.3% [32]. In the study area 50.5% of participants believe that TMs can cure some diseases that cannot be treated by modern medicine which is lower than studies done in Shopa Bultum Southwest Ethiopia which is 79.47% as modern health practitioners believed in the importance of traditional medicine practitioners maintaining sufficient healthcare services to the community [32]. This might be due to the fact that the studied population highly relied on traditional medicine whereas in the study in Debre Tabor Town modern health practitioners did not rely on traditional medicine [31].

In this study 87.6% of participants reported that health education about risks and benefits of traditional medicines is important, and the finding is consistent with previously reported studies done in Merawi Town, Northwest Ethiopia, which is 90.3% [28]. 80.8% of participants reported that nonsterile preparations of traditional medicines are harmful when given by injection, and the finding is inconsistent with previously reported studies done in Merawi Town, Northwest Ethiopia, which is 96.7% [28]. And survey conducted in shirka district, Arsi zone, showed that 84% of modern health practitioners support integration of modern and traditional medical system to improve healthcare coverage of the country [31]. In this study, 69.7% of the participants reported that traditional medicines are accessible with an affordable cost in the community, and the finding is consistent with previously reported studies done in Shopa Bultum, Southeast Ethiopia, which showed that 71.52% of the respondents prefer traditional medicine in comparison to modern medicine due to affordability, accessibility, and acceptability by the community [31].

The study illustrates that 35.8% of the community were seeking both self-medication using traditional medicine and visiting traditional medicine practitioners to manage their acute or chronic illnesses. This result is inconsistent with a study done in Shopa Bultum, Southeast Ethiopia (72.85%) and in shirka district, Arsi zone, which showed that about 79% of the modern health practitioners have visited traditional healers at least once in their lifetime to seek treatment [30, 31]. This dissimilarity may be due to differences in the cultural acceptability of healers, the respect they have, and their easy accessibility to the client's hierarchy of modern medicine.

In this study, 22.6% of the participants had positive attitudes towards the integration of traditional medicine and modern medicine which is very lower than the study done in Shopa Bultum, Southeast Ethiopia, 92% [32].

In the studied area, some aliments treated by traditional medicine include abdominal cramp, abortion, burn, chest pain, cold, cough, cutaneous leishmaniasis, diarrhea, dry cough, fungal infection, gastritis, goiter, hemorrhoid, herpes zoster, hypertension, impotence, infected wound, joint pain, liver disease, malaria, myositis, rabies, scabies, tapeworm, tonsillitis, and toothache. Of these aliments, 90.91% were infectious diseases and the rest, 9.09%, were either acute or chronic noninfectious diseases. This result is consistent with a previously reported study in Shopa Bultum, Southeast Ethiopia [31]. This similarity might be due to the evolution of curative practices that closely follow the path of the diseases.

According to the results of this study, 53.2% of study participants believe that breaking the secrecy of traditional medicines may lead to the loss of its effectiveness. The value is relatively higher than the study done in Shopa Bultum, Southeast Ethiopia, which was 35.76% [32]. This might be due to the income and psychological purposes. Some healers believe that if secrecy is broken, the treatment loses its efficacy. And the payment contributes to the efficacy of the treatment.

From our study elderly participants showed significant difference from ages 18–27 years (*P*=0.040, OR = 4.283, CI = 1.070–20.03) and from ages 28–37 years (*P*=0.033, OR = 4.283, CI = 4.793–20.281) regarding knowledge about traditional medicine. Female participants showed significant differences (*P*=0.037) regarding knowledge about traditional medicine compared to male participants. Participants with educational status of college diploma and above showed a significant difference (*P* < 0.001) about knowledge of traditional medicine compared to those participants who cannot read and write and from grades 1 to 6 students. Participants who follow Christian religion showed significant difference (*P*=0.023, OR = 3.649, CI = 1.191–11.176) about knowledge of traditional medicine compared to participants who follow Muslim religion. This may be associated with the number of Muslim participants which were 3.6% of the total included participants which is very small compared to Christian participants (96.4%). Married participants showed significant difference (*P*=0.015, OR = 2.497, CI = 1.191–5.236) about knowledge of traditional medicine compared to other groups of participants. Participants with educational status of college diploma and above showed a significant difference (*P*=0.001, OR = 3.080, CI = 1.608–5.899) about the attitude of traditional medicine compared to other groups of participants. Elderly participants showed significant difference (*P*=0.029, OR = 5.362, CI = 1.189–24.174) about utilization of traditional medicine compared to other groups of participants. Participants with educational status of college diploma and above showed a significant difference (*P*=0.002, OR = 3.080, CI = 1.608–5.899) about the utilization of traditional medicine compared to other groups of participants. Widowed participants showed significant difference (*P*=0.021, OR = 2.794, CI = 1.166–6.698) about knowledge of TM compared to single participants (*P*=0.032, OR = 2.148, CI = 1.070–4.311) and compared to married participants about utilization of TM.

## 5. Conclusions

The population has good knowledge about the traditional medicine but poor attitude and practice despite the acceptability, easy accessibility, and affordability of traditional medicine in the communities of Debre Tabor town. Age, sex, educational status, and marital status showed significant difference in knowledge; educational status showed significant differences in attitude and age, educational, and marital status showed significant difference in utilization of traditional medicine.

## 6. Recommendation

The communities in the study area have good knowledge but a lower attitude and practice in traditional medicine. They need continuous education on benefit, availability, efficacy, safety, mode of preparation, storage, and use of traditional medicine. About 53.2% of participants believed that breaking the secrecy may lead to loss of effectiveness and prefer keeping their knowledge as a secret unless necessary measures are taken; the useful traditional medicines may be lost due to lack of responsible and more trained human power. In addition to this, focus should be given to traditional medicine knowledge to promote their use and research should be encouraged on the issue.

Fifty percent of participants believed that traditional medicines can cure some diseases that cannot be treated by modern medicines. This is a potential area to develop effective medicines used to treat diseases that cannot be treated by currently available modern medicines so further in-depth studies should be encouraged. Thus, documentation of traditional medicine should be encouraged to preserve knowledge, attitude, and practice of traditional medicines.

## Figures and Tables

**Table 1:** Sociodemographic characteristics of study participants in the communities of Debre Tabor Town, North Central Ethiopia, 2018.

Characteristics of study participants	Number	Percent
Sex		
Male	297	73.13
Female	108	26.87

Age (years)		
18–27	97	24.1
28–37	168	41.8
38–47	71	17.7
48–57	39	9.7
58–67	19	4.7
>67	8	2

Educational level		
Who cannot read and write	49	12.2
Who can read and write	40	10
From grades 1–6	28	7
From grades 7–12	27	6.7
Technical and vocational certificate	79	19.7
College diploma and above	179	44.5

Religion		
Christian	388	96.4
Muslim	14	3.6

Occupation		
Government employees	162	40.3
House wife	91	22.6
Private-sector employees	83	20.6
Merchants	43	10.7
Students	23	5.6

Family monthly income		
200–2000	247	61.4
2001–3800	132	32.8
3801–6000	18	4.5
>6000	5	1.2

Marital status		
Married	298	74.1
Single	56	13.9
Widowed	36	9
Separated/divorced	12	3

Family size		
1-2	161	40
3-4	154	38.3
5-6	72	17.9
>6	15	3.7

From the total of 402 study participants, 322 (80.1%) of the participants had good knowledge about traditional medicines (see Table 2).

**Table 2:** Knowledge of study participants about traditional medicines in the communities of Debre Tabor Town, North Central Ethiopia, 2018.

Variables	Attributes	Number	Percent
Have you ever heard of TM?	Yes	327	81.3
	No	75	18.7
Are traditional medicines accessible with affordable cost in the community?	Yes	280	69.7
	No	122	30.3
Is health education about risks and benefits of TMs important?	Yes	352	87.6
	No	50	12.4
Are nonsterile TMs harmful when given by injection?	Yes	325	80.8
	No	77	19.2
Are traditional medicines more effective and safer than modern health services?	Yes	127	31.6
	No	275	68.4
Do traditional medicines produce less adverse effect compared to MM?	Yes	306	76.1
	No	96	23.9
Overall knowledge of participants	Good knowledge	322	80.1
	Poor knowledge	80	19.9

TM = traditional medicine and MM = modern medicine. From the total of 402 study participants, 158 (39.3%) of the participants had good attitude about traditional medicines (see Table 3).

**Table 3:** Attitude and practice of study participants on traditional medicines in the communities of Debre Tabor Town, North Central Ethiopia, 2018.

Variables	Attributes	Number	Percent
Do you recommend the use of TM in the community?	Yes	133	33.1
	No	269	66.9
Do you believe that TMs are still accepted and available with an affordable cost in the community?	Yes	127	31.6
	No	275	68.4
Do you believe that breaking secrecy of TMs may lead to loss of its effectiveness?	Yes	214	53.2
	No	188	46.8
Do you support integration of MM with TM to improve healthcare coverage?	Yes	91	22.6
	No	311	77.4
Do you believe that TMs can cure some diseases that cannot be treated by MM?	Yes	203	50.5
	No	199	49.5
Do you think that if TMs are formulated in a modern dosage form, it will be good enough to treat diseases with an appropriate dose and route?	Yes	335	83.3
	No	67	16.7
Do you have plans to use TM in the future?	Yes	135	33.6
	No	267	66.4
Overall attitude of participants	Good attitude	158	39.3
	Poor attitude	244	60.7

TM = traditional medicine and MM = modern medicine. From the total of 402 study participants, 145 (36.1%) of the participants used traditional medicines in their lifetime and from these participants 41 (28.3%) encountered minor adverse effects (see Table 4).

**Table 4:** Practice of study participants on traditional medicines in the communities of Debre Tabor Town, North Central Ethiopia, 2018.

Variables	Attributes	Number	Percent
Have you used herbal medicines either by yourself or through visiting a traditional healer at least once in your lifetime for treatment?	Yes	144	35.8
	No	258	64.2
Have you encountered any adverse effects?	Yes	42	29.0
	No	103	71.0

**Table 5:** Medicinal plants used for the treatment of human diseases: local name, scientific name, disease treated, part(s) used, dosage form, method of preparation, administration route, duration of use, and sources of medicinal plants where it is collected.

Local name (Amharic)	Scientific name	Disease treated	Parts used	Dosage form	Method of preparation, route, frequency of administration, and duration of use	Source of plant
Adirqit		Burn	Leaf	Solid	Squeezing juice and apply topically once daily for seven days	Garden
Anfar	*Buddleja polystacha*	Goiter	Leaf	Liquid	Maceration taken orally as a single dose	Wild plant
Anfar	*Buddleja polystacha*	Infected wound	Root	Solid	Grind into powder, mix with butter, and insert under the skin; leave for one week and remove	Wild plant
Anfar	*Buddleja polystacha*	Malaria	Root	Liquid	Maceration/decoction taken orally once daily for seven days	Wild plant
Astenagir	*Datura stramonium*	Fungal infection	Leaf	Liquid	Squeeze juice and apply topically on the affected area once daily until healed	Wild plant
Atuch	*Verbena officinalis*	Chest pain	Leaf	Liquid	Prepare as tea, taken orally as a single dose	Wild plant
Azohareg	*Clemantis hirsuta*	Cutaneous leishmaniasis	Leaf	Solid	Grind into powder, mix with butter, and apply topically once daily for two days	Wild plant
NechBahirzaf	*Eucalpytus globulus*	Cold	Leaf	Gaseous	Boil with water, cover the body with clothing, and inhale the vapor once daily for two days	Garden
Bisana	*Croton macrostachyus*	Herpes zoster	Bark	Solid	Grind into powder, mix with butter, and apply topically once daily for seven days	Garden
Bisana	*Croton macrostachyus*	Malaria	Leaf	Liquid	Macerate with water; take two doses orally for one day	Garden
Bisana	*Croton macrostachyus*	Infected wound	Leaf	Solid	Grind into powder, mix with honey, and apply topically once daily for seven day	Garden
Bisana	*Croton macrostachyus*	Fungal infection	Leaf	Liquid	Cut the base of the leaf; apply topically the watery fluid once daily until healed	Garden
Buna	*Coffea arabica*	Infected wound	Fruit	Solid	Grind into powder, mix with water, and apply topically once daily until healed	Market
Buna	*Coffea arabica*	Cough	Leaf	Solid	Chew fresh leaves once daily until cough disappears	Garden
Chegogot	*Alternana theranodiflora*	Liver disease	Leaf	Solid	Grind into powder, boil in water, and inhale vapor once daily for seven days	Wild plant
Chifirig	*Gomphocarpus stenophylus*	Impotence	Root	Liquid	Maceration, taken orally once daily for seven days	Wild plant
DamaKese	*Ocimum lamiifolium*	Cold, mich, and tonsillitis	Leaf	Liquid	Maceration/decoction, taken orally once daily until improved	Garden/field
DamaKese	*Ocimum lamiifolium*	Fungal infection	Leaf	Liquid	Squeeze juice and apply topically once daily 7 days	Garden/field
Digita	*Calpurnia aurea*	Malaria and infected wound	Leaf	Liquid	Maceration, taken orally once daily for seven days	Garden/Field
Duba	*Proteagaguedi*	Tapeworm	Seed	Solid	Roasting, taken orally as single dose	Garden
Enbacho	*Rumex nervosus*	Haemorrhoid	Stem	Solid	Grinding to powder, mix with butter and apply topically once daily until heal	Wild plant
Enbacho	*Rumex nervosus*	Burn and scabies	Leaf	Solid	Grinding to powder, mix with butter and apply topically once daily until heal	Wild plant
Enbuay	*Solanum incanum*	Myoscitis (lifi)	Leaf	Solid	Grinding to powder, mix with butter and apply topically once daily until heal	Wild plant
Endod	*Phytolaca dodecandra*	Malaria	Root	Liquid	Maceration, taken two doses orally for one day	Wild plant
Endod	*Phytolaca dodecandra*	Abortion	Leaf	Liquid	Maceration, taken single dose orally	Wild plant
Endod	*Phytolaca dodecandra*	Cough	Root	Liquid	Prepare as tea taken orally 2x/day for two days	Wild plant
Endod	*Phytolaca dodecandra*	Liver disease	Leaf	Solid	Grinding to powder, boil in water inhale once daily for 7 days	Wild plant
Enqoqo	*Embelia schimperi*	Tapeworm	Seed	Liquid	Grinding to powder, mixed with beer taken orally as single dose	Garden
Eret	*Aloe pulcherrima*	Abdominal cramp	Stem	Liquid	Maceration, taken single dose orally maceration	Wild plant
Feto	*Lepidium sativum*	Abdominal cramp	Seed	Solid	Grind into powder, mix with injera, and consume once	Garden
Gesho	*Rhamnus prinoides*	Tonsillitis	Leaf, fruit	Liquid	Maceration, taken single dose orally	Garden
Gibto	*Lupinus albus*	Hypertension	Seed	Solid	Roast, soak in water, and mix with pepper and consume 2x/day as needed	Garden
Gimero	*Cappartus tomentosa*	Infected wound	Root	Solid	Grinding to powder, mix with butter and apply topically once daily for 7 days	Garden
Girawa	*Vernonia amygdalina*	Liver disease	Leaf	Solid	Grind into powder, boil in water, and inhale vapor once daily for seven days	Wild plant
Gorjejit	*Hygrophila schulli*	Infected wound	Leaf	Solid	Grind into powder, mix with butter, and apply topically once daily until healed	Wild plant
Gorteb	*Plantago lanceolata*	Infected wound	Leaf	Solid	Grind into powder, mix with butter, and apply topically once daily until healed	Wild plant
Haregresa	*Zehner iascarba*	Rabies, michi and joint pain	Leaf	Liquid	Prepare as tea; take orally as a single dose	Garden
Haregresa	*Zehneria scarba*	Fungal infection	Leaf	Liquid	Squeeze juice and apply topically once daily until healed	Wild plant
Kebericho	*Echnopskebericho*	Malaria	Root	Liquid	Maceration; take orally once daily for seven days	Wild plant
Kutintina		Abdominal cramp	Root	Solid	Chewing fresh root as single dose to relieve the symptom	Garden
Lomi	*Citrus aurantifolia*	Scabies	Fruit	Liquid	Squeeze juice and apply topically once daily until healed	Garden
Mekimeko	*Rumex abyssinicus*	Diarrhea	Root	Liquid	Maceration; take orally once daily until healed	Wild plant
Nech shinkurit	*Allium sativum*	Cold, gastritis, malaria	Bulb	Solid	Chop into pieces and take orally once daily for seven days	Garden
Qil		Chest pain	Leaf	Gaseous	Boil in water and inhale vapor once daily for three days	Garden
Quandiro		Infected wound	Bark	Solid	Grind into powder and apply topically once daily until healed	Wild plant
Senafich	*Brassica nigra*	Cold	Seed	Solid	Grind into powder, boil in water, and inhale vapor	Garden
Simiza	*Justicias chimperiana*	Liver disease	Leaf	Liquid	Maceration with water and take orally 3x/day for weeks	Garden
Tenadam	*Ruta chalepensis*	Cold	Leaf	Liquid	Prepare as tea and take orally as needed	Garden
Tinbaho	*Nicotiana tabacum*	Toothache	Leaf	Gaseous	Boil in water and inhale vapor at the evening until improvement	Wild plant
Tinjut	*Otostega integrifolia*	Abdominal cramp	Root	Solid	Chewing the root as needed	Wild plant
Tinjut	*Otostega integrifolia*	Lemich	Leaf	Gaseous	Put on fire and fumigate daily for three days	Wild plant
Tosign	*Satureja punctata*	Hypertension and cold	Leaf	Liquid	Prepare as tea and taken orally as needed	Garden
Wanza	*Cordia africana*	Burn	Leaf	Solid	Grinding into powder, mix with butter, and apply topically once daily until healed	Garden
Wanza	*Cordia africana*	Diarrhea	Root bark	Liquid	Maceration and take orally once daily until healed	Garden
Wonberet		Tenea capitis	Leaf	Solid	Grind into powder, mix with butter, and apply topically once daily until healed	Wild plant
Wonberet		Infected wound	Leaf	Solid	Grind into powder and apply topically once daily until healed	Wild plant
Yeberemilas		Abdominal cramp	Root	Liquid	Maceration and take orally as single dose	Wild plant
Yemidirenbuay	*Solanum anguivi*	Abdominal cramp and malaria	Root	Liquid	Maceration and take orally as single dose	Garden
Zinjibil	*Zingiber officinale*	Abdominal cramp	Rhizome	Solid	Chewing and swallowing the juice as needed	Market

## Data Availability

All data generated or analyzed during this study are given in the supplementary information file.

## Supplementary Materials

Supplementary Materials KAP study data generated or analyzed during this study.
